# Body Composition Changes in Weight Loss: Strategies and Supplementation for Maintaining Lean Body Mass, a Brief Review

**DOI:** 10.3390/nu10121876

**Published:** 2018-12-03

**Authors:** Darryn Willoughby, Susan Hewlings, Douglas Kalman

**Affiliations:** 1 Department of Health, Human Performance and Recreation, Baylor University, Waco, TX 76798, USA; darryn_willoughby@baylor.edu; 2 Department of Nutrition, Central Michigan University, Mount Pleasant, MI 48859, USA; 3 Substantiation Sciences Inc., Weston, FL 33332, USA; dkalman@nova.edu; 4 HPD Exercise Science, Nova Southeastern University, Davie, FL 33314, USA

**Keywords:** weight loss, lean body mass, weight loss strategies, dietary supplements, chromium picolinate

## Abstract

With over two-thirds (71.6%) of the US adult population either overweight or obese, many strategies have been suggested for weight loss. While many are successful, the weight loss is often accompanied by a loss in lean body mass. This loss in lean body mass has multiple negative health implications. Therefore, weight loss strategies that protect lean body mass are of value. It is challenging to consume a significant caloric deficit while maintaining lean body mass regardless of macronutrient distribution. Therefore, the efficacy of various dietary supplements on body weight and body composition have been a topic of research interest. Chromium picolinate has been shown to improve body composition by maintaining lean body mass. In this paper we review some common weight loss strategies and dietary supplements with a focus on their impact on body composition and compare them to the effect of chromium picolinate.

## 1. Introduction

Over one-third (36.5%) of the US adult population are obese as categorized by having a body mass index (BMI) ≥ 30 kg/m^2^ [1]. The most common causes of obesity are overeating and physical inactivity. However, ultimately, body weight and its composition are the result of genetics, metabolism, environment, behavior, and culture. It is well recognized that obesity not only has a significant, adverse impact upon disease risk, but also has important consequences for morbidity, disability, emotional well-being, and quality of life. Therefore, efforts to prevent, reduce or intervene in weight gain and obesity are at the forefront of public health concerns.

Due to the great cost to both physical and psychological health, an abundance of clinical studies have been conducted on various interventions to improve body weight and composition. Focusing on body composition is essential because even more important than overall weight loss is sustainably losing fat mass (FM) while maintaining lean body mass (LBM). Most popular commercial weight loss programs are marketed as being able to reduce body weight within the first few weeks; however, of the weight lost, a significant amount includes losses in both LBM and FM, as well as changes in fluid status. Much of the early research utilized weight loss interventions that were designed around dietary changes that focused on calorie restriction and outcomes focused primarily on total body weight changes as opposed to body composition changes [2]. This approach was found to be misleading and potentially detrimental to health due to the fact that substantive reductions in LBM also occurred and weight lost was often quickly regained [3].

The loss of LBM is problematic for a number of reasons, including impacts on health, ability to conduct activities of daily living and potential effects on emotion and psychological states. The loss of LBM impedes sustainability by causing lowered resting energy expenditure/metabolism, fatigue, declines in neuromuscular function, and increased risk for injury [4,5]. Moreover, the metabolic decline that occurs after LBM loss results in a subsequent body fat overshoot, or a regain in fat mass [6], thereby resulting in unfavorable, compounded changes in body composition. Therefore, in order to enhance sustainability of any weight loss program, and to offset any potential negative health consequences, it is important to prevent the loss of LBM. In an effort to offset this LBM loss, several studies have incorporated exercise in conjunction with a weight loss program; however, this has only provided modest outcomes, most likely due to the modest caloric expenditure experienced with moderate exercise [7]. Various dietary supplement products have also been researched to augment weight loss or improve body composition with varying levels of success. Some well-studied weight loss and body composition dietary supplements include, but are not limited to, fiber complex, green tea, *Garcinia cambogia, Irvingia gabonensis*, and chromium picolinate.

Of these products, chromium picolinate (Chromax^®^, Purchase, NY, USA; CrP) has been shown to help individuals lose fat and overall body weight while also preserving lean muscle mass. It has been reported that supplementation with 200–400 mcg chromium/day as chromium picolinate improves body composition [8], which is an important aspect for overall metabolic health and, therefore, weight loss and maintenance. Dietary chromium is an essential nutrient with recommended daily intakes of 50–200 mcg chromium/day. It has been shown to play an important role in glucose, lipid, and amino acid metabolism by its potentiating effects on insulin action [9]. However, the dietary intake of chromium is typically suboptimal as few diets contain even the minimum of 50 mcg [9]. Since most people consume half of the recommended amount, the likelihood of chromium supplementation being beneficial is increased. Therefore, adding bioavailable forms of chromium to the diet is vital for individuals to benefit from the effects of chromium on body composition. 

To increase the bioavailability of chromium, it was added to picolinic acid, a naturally occurring metabolic derivative of tryptophan. Combining picolinate acid with chromium in the form of chromium picolinate (CrP) enhances the bioavailability of chromium compared to other salts of chromium, and by doing so improves insulin utilization and efficiency in humans and animals [10]. Many studies which have reported beneficial effect of CrP have been performed in preclinical models. Improving insulin utilization can positively influence body composition because of the role insulin plays in the endogenous synthesis of fatty acids and triglycerides, as well as increasing muscle protein synthesis [11,12]. CrP has been extensively studied for its ability to enhance body composition and in over thirty-five human clinical trials has been shown to enhance weight loss, caloric reduction, and carbohydrate and glucose metabolism, among various other benefits. Not all studies report weight loss with chromium supplementation, including a systematic review of overweight and obese subjects that had a narrowed search criteria to studies focusing on weight loss, not LBM or body composition changes, which is focused on in this paper [13]. In a key clinical study, CrP supplementation not only lead to weight loss, but also favorable body composition changes, as the weight lost was 98% FM and only 2% LBM [14]. 

Based on the understanding that the decline in LBM during weight loss can negatively affect various physiological processes and in turn, hinder weight loss maintenance and healthy body composition, the purpose of this brief review is to evaluate the impacts of various dietary strategies on LBM as well as popular dietary supplements often used as adjuncts to improve weight loss outcomes including maintenance of LBM. Pub Med and Google Scholar were searched for the following terms: weight loss and lean body mass; weight loss and body composition; as well as weight loss and lean body mass associated with each supplement and dietary strategy covered.

## 2. Weight Loss Strategies

There are many different weight loss strategies with many sub types and commercial plans. Most include some sort of caloric restriction and typically focus on a specific macronutrient range. Based on the problematic loss of LBM associated with typical weight loss regimens, recent weight loss studies have focused not just on total weight lost, but also on body composition changes in addition to total weight lost [15]. 

**Figure 1:**
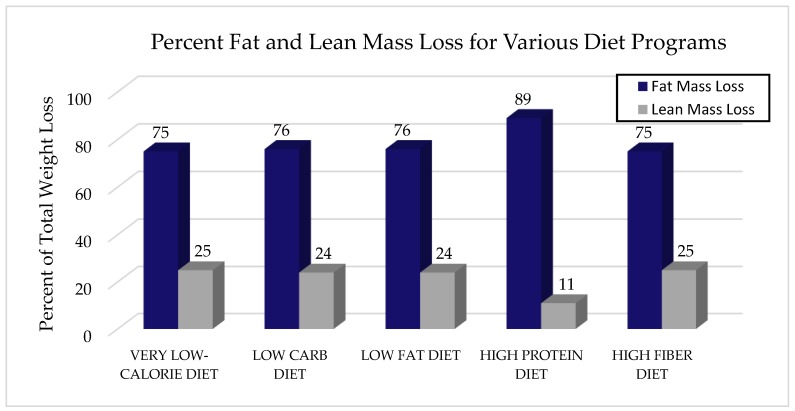
An overview of various weight loss clinical trials to examine the changes in body composition that occur from using popular diet programs. It should be emphasized that each diet program is distinct and contains unique and different exercise programs and diets that are thought to be helpful for weight loss but are not always evaluated by change in body composition. Results showed that all the popular diet programs examined lead to weight loss, though a large percent of the weight lost during these diet programs comes from a loss in LBM [14,16,17,18].

Following are a summary of popular diets focusing on the results in body composition changes including the very low-calorie diet, the ketogenic diet which strictly limits carbohydrates and focuses on fat, the high protein diet which limits carbohydrates and focuses on protein and a high fiber diet which focuses on high-fiber carbohydrates, see Figure 1 comparing these diets.

### 2.1. Very Low-Calorie Diet

The very low-calorie diet (VLCD) approach is typically used to achieve rapid weight loss and is centered on meal replacement powders and ready to drink beverages. A VLCD typically includes 400–800 kcalories (kcal)/day and is intended for rapid weight loss [15]. Although the weight loss observed with VLCD treatment is clinically significant, the accompanying decline in LBM may be equally significant and therefore detrimental. For instance, overweight subjects (*n* = 127) who underwent a VLCD (430 kcal/day) for eight weeks lost a mean of 12.7 kg of total weight, of which 75% was fat loss and 25% was LBM loss [16]. A recent study using a popular VLCD system (Optifast^®^, Nestlé HealthCare Nutrition, Bridgewater, NJ, USA) supplemented with whey protein described a total body weight loss of 17 kg, of which 4.6 kg was LBM (approximately 25% of the weight lost), after twelve weeks of following this 1120 kcal/day, high protein, low-fat diet [7]. Historically, fast and overall weight loss was focused on and valued, but more current thought is that the focus should be on body composition changes as opposed to solely judging success by total weight loss. Based on this data it is evident that a VLCD leads to a large initial drop in total weight loss, but also a great loss in LBM, and therefore might not be the best option for sustainable weight loss and body composition improvements.

### 2.2. Ketogenic Diet

The rationale for this diet is that low-carbohydrate conditions lead to skeletal muscle lipolysis and the subsequent release of fatty acids into the circulation, bound to albumin. Low-carbohydrate diets that induce a state of ketosis (“ketogenic” diet) have been explored in relation to changes in body composition. Ketosis forces the body to burn fats rather than carbohydrates. For example, Frisch et al., examined changes in body composition after enrolling two hundred subjects in either a low-carbohydrate or low-fat diet program for 12 months. Study results showed that both diets resulted in similar amounts of weight loss (5.8 kg vs. 4.3 kg, respectively; *p* = 0.065), with 76% of the weight loss from FM and 24% from LBM loss in the low-carbohydrate group [17]. Another study determined whether a four-week isocaloric low-carbohydrate, ketogenic diet or a high-carbohydrate, baseline diet was associated with changes in body composition in overweight or obese men. Compared with the baseline diet, the ketogenic diet was not accompanied by increased body fat loss but coincided with increased protein utilization and loss of fat-free mass [19]. Another study evaluated the changes in body composition of obese individuals following a very low-calorie ketogenic diet for four months. After four months, the ketogenic diet induced weight loss mainly at the expense of FM and visceral FM, while preserving LM [20]. A recent review of 13 ketogenic diet studies described reductions in total body weight of 5–13 kg and accompanying decreases in LBM of 1–3.5 kg (approximately 20–25% of weight lost was from LBM) [21].

### 2.3. High Protein Diet

Protein intakes above the recommended 0.8 g protein/kg/day, or 10–35% of total calories [22], are often suggested as a strategy to offset the loss of LBM experienced with caloric restriction. This is due primarily to the role protein, especially the amino acid leucine, plays in inducing muscle protein synthesis and, to some extent, in increasing satiety [23]. Research suggests that ≥2 g protein/kg body weight/day may be required to maintain LBM during a calorically-deficient diet [24].

During a four-week 40% energy deficient diet, overweight recreationally active men consuming 2.4 g protein/kg body weight/day experienced greater increases in LBM and losses in FM than those consuming a diet containing 1.2 g protein/kg body weight/day when combined with a high volume of resistance exercise [25].

The influence of different dietary compositions and a regular exercise program was evaluated in 161 obese women over 14 weeks. Subjects were assigned one of the following groups: (1) a high energy, high carbohydrate, low protein diet (HED) (2600 kcals; 55% carbs: 15% protein: 30% fats), (2) a very low carbohydrate, high protein diet (VLCHP) (1200 kcals; 63% protein: 7% carbs: 30% fats), (3) a low carbohydrate, moderate protein diet (LCMP) (1200 kcals; 50% carbs: 20% protein: 30% fats) or (4) a high carbohydrate, low protein diet (HCLP) (1200 kcals; 55% carbs: 15% protein: 30% fats) accompanied by a thrice-weekly supervised, circuit-type resistance exercise program. Collectively, these groups showed markedly greater reductions in body weight compared to women that did not reduce calorie intake but did the exercise program, but significant declines in LBM were still noted (approximately 11–23% of the weight lost) [26].

### 2.4. High Fiber Diet

It has been suggested that when comparing diets of varying macronutrient distributions, the type of carbohydrate consumed may influence results because high fiber carbohydrate sources may promote weight loss via increased satiety [18] and greater nutrient density when compared to lower fiber sources. Furthermore, high fiber intake may help to decrease caloric absorption by binding with fat [27]. A comparison of energy-reduced diets with a high protein or high fiber/carbohydrate focus found significantly greater weight and body fat decreases with the high protein-focused diet but reductions in LBM between groups was minimal. These results indicated that the subjects following the high protein diet lost more weight and body fat than those on the higher fiber diet, but they still lost LBM [28]. 

## 3. Dietary Supplements and Body Composition

**Figure 2:**
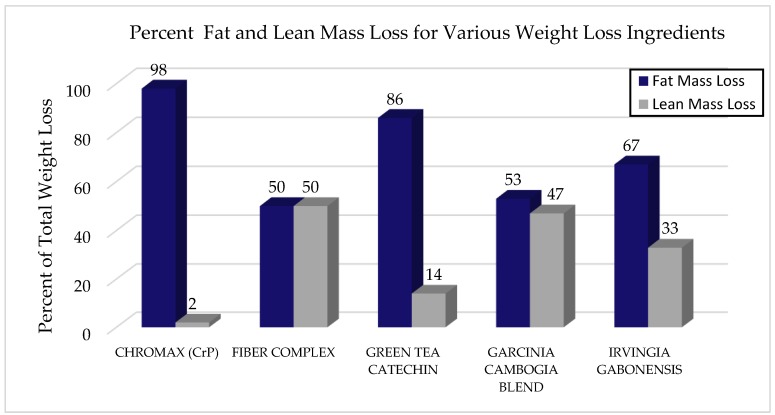
The changes in body composition that occur from using CrP versus other popular dietary supplements from various weight loss clinical trials are presented. It should be emphasized that each supplement and study conditions are distinct and contain unique and different exercise programs and diets that are thought to be helpful for weight loss, but ultimately are not evaluated by overall body composition. Results showed that compared to other popular weight loss supplements, CrP supplementation resulted in the greatest percentage of FM loss and the smallest percentage of LBM loss from total weight loss. While CrP and other dietary supplements all lead to weight loss, a larger percent of the weight lost during these studies comes from a loss in LBM. CrP appears to offer an effective means of improving body composition, as individuals are able to lose their fat, while retaining their muscle [14,31,32,33,34].

Exercise has been shown to help offset some of the changes in LBM experienced with weight loss [29,30]. However, exercise is not always feasible or sustainable for everyone and may not always offset loss of LBM to an extent that attenuates the negative impact on health. Therefore, because of the challenge of preventing significant LBM loss in many weight loss programs, additional research has examined the efficacy of various dietary supplements on body weight and body composition. Figure 2 illustrates various studies that have been conducted with selected dietary supplements on FM loss versus LBM loss in comparison to CrP. Dietary supplements were chosen for their popularity as well as the availability of research looking at FM and LBM loss. 

### 3.1. Chromium Picolinate

Chromium is an essential trace mineral involved in carbohydrate, fat and protein metabolism and is combined with picolinic acid to enhance absorption. It facilitates the action of insulin, has been shown to improve glycemic control in diabetes [35], and has been shown to improve body composition by helping to maintain LBM [36]. Moreover, enhanced insulin action increases the rate of glucose and amino acid uptake in muscle cells. 

Several studies support its role in maintaining LBM during weight loss [8,14,31,37,38]. In one study, obese subjects followed a VLCD for eight weeks followed by 18 weeks of weight maintenance and were supplemented with 200 mcg of chromium from CrP; they showed an increase in LBM compared to those following the same weight loss program but taking placebo or chromium yeast (*p* < 0.029) [37]. Moreover, Kaats et al., ported a statistically significant improvement in body composition after 90 days of supplementation with 400 mcg/day of chromium from CrP in 122 subjects who continued with their normal physical and dietary habits. CrP supplementation not only lead to weight loss, but also favorable body composition changes, as the weight lost was 98% fat mass and only 2% LBM [14]. 

CrP at a 400 mcg of chromium dose taken for 24 weeks by competitive swimmers significantly increased lean mass by 3.5%, decreased FM by 4.5%, and decreased body fat percentage by over 6% when compared to the placebo group [38]. Moreover, increases in LBM have been experienced in subjects not attempting weight loss but taking 200 mcg of chromium from CrP [8,31]. Kaats et al., reported a statistically significant improvement in body composition after 72 days of supplementation with either 200 mcg/day or 400 mcg/day of chromium from CrP in 154 subjects not attempting weight loss. While there was no difference between those taking 200 mcg and those taking 400 mcg, both showed significantly greater improvement than those taking a placebo [8]. 

### 3.2. Fiber Complex

Fiber has been explored as a weight loss aid both as a dietary component and as a dietary supplement. It contributes to satiety [32] and nutrient density as well as binds with fats, thereby decreasing caloric absorption. The fat binding capacity of a natural fiber complex, IQP G-002AS, derived from *Opuntia ficus-indica*, enriched with additional soluble fiber from *Acacia* spp. has been shown to reduce dietary fat absorption up to 27% in animal and human studies, and in vitro models [27]. A 12-week study with obese women and men assessed the fiber complex derived from *Opuntia ficus-indica* and *Acacia* species. The group assigned to the fiber supplement lost significantly more body weight and body fat than placebo. There were no differences in LBM, though both groups did lose LBM suggesting the fiber may have contributed to increased weight loss in the treatment group but did not effectively offset LBM losses in this study; from the total weight lost, the fiber group lost approximately 50% of LBM while the placebo group lost approximately 36% of LBM showing that the fiber did not offset LBM loss [39]. In a similar study conducted over six months, subjects maintained weight loss, but also lost LBM when compared to controls [33].

### 3.3. Green Tea

Green tea (*Camellia sinensis*) extract, comprised of catechins and caffeine, has been the subject of significant investigations related to body weight [40,41]. Green tea extract (GTX) enriched beverages, differing by catechin and caffeine content and serving frequency, were compared for their effects on body weight and body composition in moderately overweight subjects for 90 days without any calorie restriction. Daily consumption of a GTX drink providing 886 mg catechins and 198 mg caffeine/day resulted in the greatest percent decrease in intrabdominal and total fat mass and total body mass, attended by a slight decline in LBM [40]. Similarly, daily consumption of a beverage containing 625 mg catechins and 39 mg caffeine/day for 12 weeks resulted in greater total weight loss and fat mass loss in the abdominal area compared to placebo. Of the total weight lost, 86% came from FM and 14% came from LBM loss [41].

### 3.4. Garcinia Cambogia

*Garcinia cambogia* (Malabar tamarind) is a fruit native to Southeastern Asia. The rind is commonly used as a food preservative, flavoring agent or food-bulking agent, and as a traditional remedy for constipation, edema and many other common ailments [34]. Studies have suggested that it has anti-obesity, hypolipidemic, and many other functions, but it is best known and sold for its role in weight loss [34]. *Garcinia cambogia* supplements contain between 20 to 60% hydroxycitric acid (HCA). HCA is the key component from the rind that may be responsible for weight loss effects [42]. In vivo studies confirmed the role of *G. cambogia*/HCA in stimulating fat oxidation, increasing serotonin release in the brain cortex and normalizing lipid profiles in humans. Clinical studies in obese subjects indicate that it may aid in weight loss via its role in increasing serotonin levels [34]. Safety is well established when the extract is used alone; however, in efficacy studies it is often mixed with other components in weight loss formulas. For example, a supplement comprised of standardized extracts of *Garcinia cambogia*, *Camellia sinensis*, unroasted *Coffea arabica* (green coffee fruit), and *Lagerstroemia speciosa* (banaba leaf) was provided in a clinical study among 92 overweight subjects following a moderate calorie-restricted diet for 12 weeks. Although the group receiving the supplement showed a significantly greater reduction in total body fat, approximately half of the weight lost was comprised of LBM [43]. 

### 3.5. Irvingia Gabonensis

*Irvingia gabonensis* is a tree indigenous to Africa, also known as bush mango. It has been suggested that it has an antidiabetic effect due to its reported ability to decrease fasting blood sugar [44]. It has also been shown to inhibit adipogenesis in vitro [45] and to possess anticholesterol effects [46]. Moreover, there is evidence to support its role in promoting weight loss [47]. A recent systematic review concluded that “*Irvingia gabonensis* supplementation causes significant reductions in body weight and waist circumference.” However, the authors questioned the quality and duration of the studies conducted and would not recommend supplementation until further well controlled studies are conducted [48]. In a randomized, double blind, placebo-controlled crossover design, 40 obese subjects were given two different types of capsules containing 350 mg of *Irvingia gabonensis* seed extract (active formulation) or oat bran (placebo) for four weeks. Three capsules were taken three times daily, one-half hour before meals (a total daily amount of 3.15 g of *Irvingia gabonensis* seed extract) with a glass of warm water. Subjects supplementing with the extract achieved a significantly greater decrease in total weight, 2.9% after two weeks and 5.6% after one month (*p* < 0.0001). Percent body fat was not significantly reduced, suggesting the weight lost came from a loss of LBM [49]. 

## 4. Conclusions

It is well recognized that obesity not only has a significant, adverse impact upon disease risk but also has important consequences for morbidity, disability, emotional well-being, and quality of life. Therefore, efforts to address the issue have received much attention in the literature. While various weight loss programs have resulted in short term success, many fail to result in long term weight loss. One of the suggested reasons for this lack of long term success is the loss of LBM that occurs with the weight loss. Therefore, recent focus in the weight loss literature has been not just on total weight loss but on improving body composition. Focusing on the effects of any weight loss effort on body composition changes is essential because even more important than short term weight loss is sustainably losing FM while maintaining LBM. 

Many different programs and interventions have tried to offset the loss of LBM commonly experienced as part of hypocaloric popular weight loss programs through methods including dietary manipulation, especially augmented protein intake, exercise, and dietary supplements. There are a few dietary supplements that appear to help preserve LBM during weight loss or to reduce the loss of LBM as compared to diet alone. One such dietary supplement that appears to have this “LBM sparing” effect is chromium picolinate. For the reasons shared in this paper, it seems logical to include CrP as part of any reduced calorie (diet) plan as a means of keeping metabolically active LBM. Weight loss without CrP may be at the cost of LBM, thus, this adjunctive strategy appears worthy of further research and applied practice. A comprehensive approach, integrating evidence-guided macronutrient and calorie intake, resistance exercise, and chromium picolinate may be the most effective approach to preserving or increasing lean body mass while maximizing body fat mass reductions.
